# Metabolic and Morphological Aspects of Adaptation of Alkaliphilic *Bacillus aequororis* 5-DB and Alkali-Tolerant *Bacillus subtilis* ATCC 6633 to Changes in pH and Mineralization

**DOI:** 10.1155/2024/3087296

**Published:** 2024-07-23

**Authors:** Yuliya Maksimova, Ann Eliseeva, Aleksandr Maksimov

**Affiliations:** ^1^ Laboratory of Molecular Biotechnology Institute of Ecology and Genetics of Microorganisms Ural Branch Russian Academy of Sciences, Golev Str., 13, Perm 614081, Russia; ^2^ Department of Microbiology and Immunology Perm State University, Bukirev Str., 15, Perm 614990, Russia

## Abstract

The goal of the study is to evaluate metabolic and morphological changes of the facultative alkaliphile *Bacillus aequororis* 5-DB and the weakly alkali-resistant *B. subtilis* ATCC 6633 in a wide pH range and at different NaCl concentrations. The alkaliphile *B. aequororis* 5-DB is shown to have a broader general resistance to adverse factors (wide pH range, 50 g/L NaCl) than a weakly alkali-tolerant strain of the same genus. This alkaliphile is also shown to have a significantly greater resistance not only to high pH but also to low pH in comparison with *B. subtilis* ATCC 6633. The resistance of *B. aequororis* 5-DB to low pH was expressed in higher metabolic activity, maintenance of ΔpH, and no significant cell damage. The selected set of methods (reduction of resazurin to resorufin by cell dehydrogenases, bioluminescent method for determining ATP, AFM, and measurement of intracellular pH) allows us to adequately assess the ability of microbial cells to withstand harsh environmental factors. Nonspecific resistance of *B. aequororis* 5-DB was proven using a complex of selected methods. Tolerance to a wide range of pH and high salt concentrations may be useful for biotechnological applications of the strain.

## 1. Introduction

Soda lakes and artificial alkaline biotopes, such as soda sludge reservoirs, harbor microorganisms that are resistant to high pH and salinity. The microbial community of these environments includes extremophiles and tolerant to extreme factors organisms. Alkaliphiles require an alkaline environment to live, and their optimum pH for growth is about 10.0. Based on their preference for different environmental pH values, these microorganisms are grouped into two broad categories: alkali-tolerant organisms, which exhibit optimal growth at pH 7.0–9.0 but cannot grow at pH above 9.5, and alkaliphilic organisms, which grow optimally at pH from 10.0 to 12.0. They are divided into facultative alkaliphiles, which grow well in the neutral pH range, but with an optimum at pH 10.0 or higher, and obligate alkaliphiles, which grow optimally at pH above 10.0, but do not grow at pH below 9.0 [1].

In addition to high pH, microorganisms in alkaline ecosystems are affected by high mineralization of the environment. The consequence of high salt concentration in the medium is the loss of water from the cell and plasmolysis of the cell. Turgor pressure is the force within the cell that pushes the cytoplasmic membrane against the cell wall. To support tension, a cell maintains an intracellular osmotic pressure above that of the environment [2]. To do this, halophiles carry out two strategies: (1) “salt in,” which consists in the accumulation of molar concentrations of potassium chloride in the cell and (2) the synthesis of osmoprotectants by the cell. Weak halophiles grow optimally on the media containing 0.5% to 3% salt; moderate halophiles, 3% to 15% salt; and extreme halophiles, 15% to 30% salt. In addition, many halotolerant microorganisms grow both without the addition of salt and at a salt concentration of more than 12% [3]. Survival and growth of *Bacillus subtilis* in an osmotically changing environment depends on adaptive reactions that are either part of the general stress response or specific to osmotic stress. Specific stress responses of many *Bacillus* spp. include the synthesis and absorption of certain organic osmolytes, in particular, proline, glycine betaine, and ectoine, under hyperosmotic conditions [4].

Most *Bacillus* spp. are moderately halotolerant and can grow under salinity over the 60 g/L. Thus, *B. spizizenii* was able to grow at mineralization above 57 g/L but with a long growth retardation time [5]. However, the adaptive responses of bacilli during growth at high levels of mineralization have not been studied. Zhang et al. studied morphological changes during adaptation of *Bacillus* sp. YM1 to increased salt concentrations. It was shown that in a medium with 30–50 g/L salt, the cell surface was strongly wrinkled, sticky secretions appeared on the cell surface, and sporulation increased, but the shape and size of the cells did not change significantly [6]. The adaptation of alkaliphilic bacilli has been studied in detail, mainly from the point of view of energetics. Alkaliphiles demonstrate multiple adaptive responses that allow them to live at high pH [7]. When growing in an alkaline environment with a low proton content and a high sodium ion content, alkaliphilic microorganisms, in addition to osmoadaptation, need to maintain intracellular pH homeostasis. In an alkaline environment, the formation of a chemiosmotic reverse pH gradient (∆pH) is provided by an electrochemical Na^+^ gradient using Na^+^/H^+^ antiporters, which are involved in the replacement of sodium ions with protons [8]. It was reported that in the facultative alkaliphile *Bacillus pseudofirmus* OF4, ATP synthesis occurs faster at high alkaline medium than at a pH close to neutral [9]. The effect of pH has been studied mostly in terms of its impact on growth rate [10], while the morphological responses of bacilli, ATP content, and dehydrogenases activity are poorly understood. Zhang et al. studied the expression of bacilli stress genes under salt stress [6]; however, there are insufficient data on the combined effects of high salt concentrations and high pH on bacilli metabolism and morphology. In this study, we studied the effect of pH and high NaCl concentration on the metabolic intensity and ATP content of bacilli cells from both the log phase of growth and the stationary phase under starvation conditions and with the addition of glucose. In addition, changes in intracellular pH and cell morphology were studied. We hypothesized that alkaliphilic bacilli have a more pronounced tolerance to high pH and salt concentration than alkali-tolerant representatives of the *Bacillus* sp. However, our experiments showed that alkaliphilic bacilli had a greater resistance not only to high but also to low pH, which could be related to a more pronounced nonspecific resistance. The role of spore formation in the adaptation of bacilli to extreme pH values and high salt concentration was also studied, and it was shown that adaptation is provided mainly in vegetative cells.

From the soil of the soda sludge storage with a high salt content, we previously isolated and identified *Bacillus aequororis* 5-DB by 16S rRNA sequencing [11], which has similarity of 99.87% with the new species *Bacillus aequororis* M-8T isolated by Singh et al. from a marine sediment [12]. *Bacillus aequororis* 5-DB has lipase and amylase activity, and grows at pH 11 and 50 g/L NaCl [13]. Bacilli have great biotechnological potential and are used as producers of hydrogen and polyhydroxyalkanoates [14], as probiotics [15], enzyme producers [16–21], and in food fermentation [22]. For biotechnological applications, it is important that bacteria withstand extreme conditions (high or low pH, low water activity, the presence of solvents, and suboptimal temperatures), so it is important to evaluate the metabolic activity of strains under harsh conditions. We have selected a set of methods that allow us to comprehensively assess the metabolic and morphological changes in the cells of an alkaliphilic strain of bacilli, which has the prospect of biotechnological use, in comparison with a weakly alkali-tolerant collection strain of *B. subtilis* ATCC 6633. We adapted a method for determining dehydrogenase activity using the PrestoBlue HS Cell Viability Reagent dye, based on the reduction of resazurin to fluorescent resorufin, a bioluminescent method for determining the ATP content in the cell, and an AFM method for assessing the morphological changes that occur in response to changes in pH and mineralization of the medium. We also assessed intracellular pH (pH_in_) using a 5-(and-6)-carboxyfluorescein diacetate succinimidyl ether fluorescent probe and visualized sporulation by phase-contrast microscopy.

The aim of the investigation is to evaluate the metabolic intensity and morphological changes of the facultative alkaliphile *B. aequororis* 5-DB in a wide range of pH and at different concentrations of NaCl in the medium in comparison with the weakly alkali-tolerant *B. subtilis* ATCC 6633.

## 2. Materials and Methods

### 2.1. Bacterial Strains and Culture Media

*B. aequororis* 5-DB (VKM B-3610D) was isolated from the soil surface of the soda sludge storage (Berezniki Soda Plant, Perm Krai, Russia) in the medium (g/l): peptone-10, glucose-10, yeast extract-5, K_2_HPO_4_-1, Na_2_CO_3_-10, and pH 11 [11]. *B. subtilis* ATCC 6633 was cultivated in the medium (g/l): peptone-10, glucose-10, yeast extract-5, K_2_HPO_4_-1, Na_2_CO_3_-3, and pH 8. Strains cultivated in 100 ml of the media in 250 ml Erlenmeyer flasks on the orbital shaker with a platform rotation speed of 100 rpm to logarithmic or stationary growth phase at 25°C. Biomass was concentrated for 20 min at 4444 g in a 5804R centrifuge (Eppendorf, Germany). *B. aequororis* 5-DB cells were washed three times with borate buffer (pH 11), *B. subtilis* ATCC 6633 cells were washed three times with phosphate buffer (pH 8), and cell pellets were transferred into the mineral medium (g/L): KH_2_PO_4_-1.0, K_2_HPO_4_·3H_2_O-3.7; NaCl-0.5 or 50; and pH of the medium was adjusted to 3–13 pH with 1 M NaOH or HCl solution. The incubation medium did not contain any nutrients except for the experiment with the addition of glucose, and in the latter case, 1 g/L of glucose was added to the mineral medium. Bacilli cells at different pH (3–13) and NaCl (0.5 and 50 g/L) concentrations were incubated under static conditions at 25°C.

### 2.2. Assessment of Metabolic Intensity of Cells

To assess the metabolic intensity, *B. aequororis* 5-DB and *B. subtilis* ATCC 6633 cells were stained with PrestoBlue HS Cell Viability Reagent (Invitrogen, Thermo Fisher Scientific, USA). The dye of 10 µl was added to 90 µl of cell suspension in the wells of 96-well black opaque flat-bottom plate (Nunc, Denmark). After adding the dye, the plate was incubated for 10 min at 37°C, and fluorescence was measured on an Infinite M1000 Pro plate reader (Tecan, Switzerland) at *λ*_ex/em_ 560/590 nm. Conventional units of fluorescence were related to the density of the bacterial culture, measured at *λ* 600 nm (units of fluor./OD600).

### 2.3. Assessment of ATP Content

To determine the ATP concentration in cells, 1 ml of the sample was centrifuged for 5 min at 15400 g, the supernatant was removed, and the cells were destroyed with 1 mL of DMSO for 15 min. The lyophilized reagent containing firefly luciferin and luciferase (ATP reagent, ZAO BKhM ST, Moscow, Russia) was diluted with Tris-acetate buffer (pH 7.8) according to the manufacturer's protocol, and after that, additionally, it was diluted with 25-fold dilution with Tris-acetate buffer (pH 7.8) and mixed in a 1 : 1 ratio with 10-fold dilution with deionized water samples, and 100 *μ*l of the mixture was placed in the wells of a white opaque flat-bottom plate (Nunc, Denmark). Тhe luminescence intensity was measured on an Infinite M1000 Pro plate reader (Tecan, Switzerland). The ATP concentration in the samples was estimated using a calibration graph of the dependence of luminescence intensity on the known ATP concentration.

### 2.4. Assessment of the Viable Cells and Spores

The number of viable cells of *B. aequororis* 5-DB and *B. subtilis* ATCC 6633 was assessed by inoculation in the rich agar medium with pH 11 and 8, respectively. The viable cell concentration was calculated and expressed as colony-forming units per mL (CFU/mL). The number of spores was determined in CFU/ml after heating the suspension at 80°C for 10 min to inactivate vegetative cells [23].

### 2.5. Assessment of Intracellular pH

**Figure 1:**
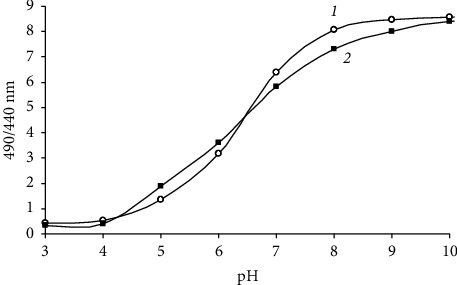
The relationship between the pH and the ratio (490/440 nm) of cFDASE in *B. subtilis* ATCC 6633 (1) and *B. aequororis* 5-DB (2). pH_in_ and pH_out_ were equilibrated by incubation with valinomycin (1 mM) and nigericin (1 mM).

Intracellular pH (pH_in_) was assessed using a 5-(and-6)-carboxyfluorescein diacetate succinimidyl ether (cFDASE) (Sigma-Aldrich, Slovakia) fluorescent probe [24]. Bacterial cultures were grown for 7 days at 25°C in the medium of the composition given above at pH 8 and 11, and cells were washed with 50 mM HEPES buffer (pH 8.0). The cell's pellets were resuspended in the same buffer. Then, the cells were incubated for 10 min at 30°C in the presence of 1.0 *μ*M cFDASE. Next, the cells were washed and resuspended in buffers with extracellular pH (pH_out_) values ranging from 3.0 to 13.0. 10 mM of glucose was added to eliminate unconjugated cFDASE, and the cells were incubated for 30 min at 30°C. Then, the cells were washed twice and resuspended in buffers with pH 3.0–13, and fluorescence was measured on Infinite M1000 Pro plate reader (Tecan, Switzerland) at excitation wavelengths of 490/440 nm and emission wavelengths of 525 nm. The extracellular fluorescence signal (background) was determined by filtering the cell suspension through a 0.22 *μ*m membrane filter, followed by measurement. Calibration curves for bacterial cultures were determined in buffers with pH values ranging from 4 to 10 (Figure 1). External and internal pH values were balanced by adding valinomycin (1 *μ*m) and nigericin (1 *μ*m).

### 2.6. Microscopy

The morphology of bacterial cells and surface profiles was studied using an Asylum MFP-3D-BIO atomic force microscope (AFM) (Asylum Research, USA) in the laboratory of atomic force microscopy and confocal microscopy at the Rhodococcus-Center of the Perm State University. Scanning was performed in the air in semicontact mode using OMCL-AC240TS-R3 silicon cantilevers (Olympus, Taiwan) coated with aluminum, with a resonant frequency of 70 (50–90) kHz, a needle curvature radius of 7 nm, and a stiffness constant of 2 (0.6–3.5) N/m. Two- and three-dimensional topographic images of bacteria were obtained to determine the linear dimensions of cells (length, width, and height) and characterize the structure of the cell surface (roughness). Preparations for AFM scanning were made immediately after placing the cells in the appropriate buffer (1-2 min) and after 24 hours. The microphotographs were processed using the Igor Pro 6.22A program (WaveMetrics, USA) [25].

Sporulation was studied using phase-contrast microscopy (S1). The native samples in the form of a crushed drop without additional staining were examined at a magnification of 1000 using a Leica DM LS microscope (Germany). Spores were distinguished from vegetative cells by the opalescence of the cell under phase contrast.

### 2.7. Statistical Analysis

The presented data are the results of three independent experiments. The results obtained were processed statistically, and the means, standard deviations, and confidence intervals were determined. The significance of differences was assessed using Student's *t*-test, *p* < 0.05 (*n* = 9).

## 3. Results

### 3.1. Assessment of the Metabolic Intensity of *B. aequororis* 5-DB and *B. subtilis* ATCC 6633 Cells Depending on the pH and NaCl Concentrations

The metabolic intensity of bacilli cells was assessed using PrestoBlue HS Cell Viability Reagent (Invitrogen, Thermo Fisher Scientific, USA). The reagent contains resazurin, which is reduced by living cell dehydrogenases to fluorescent resorufin. Bacilli were grown to the logarithmic and stationary growth phases, and the metabolic intensity was assessed after 2, 24, and 48 h in a medium with different pH and 0.5 and 50 g/L NaCl concentrations under starvation and in the presence of glucose.

**Figure 2:**
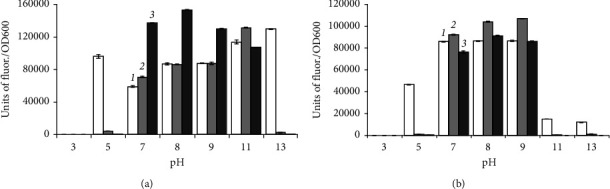
Metabolic intensity (units of fluor./OD600) of *B. aequororis* 5-DB cells from the logarithmic growth phase after 2 (1), 24 (2), and 48 (3) hours in a medium with 0.5 (a) and 50 (b) g/L NaCl.

**Figure 3:**
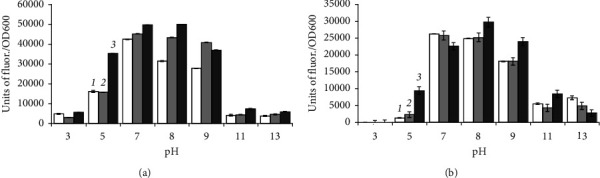
Metabolic intensity (units of fluor./OD600) of *B. subtilis* ATCC 6633 cells from the logarithmic growth phase after 2 (1), 24 (2), and 48 (3) hours in a medium with 0.5 (a) and 50 (b) g/L NaCl.

*B. aequororis* 5-DB and *B. subtilis* ATCC 6633 cells from the logarithmic growth phase were incubated in a medium with pH from 3 to 13 with two concentrations of NaCl (0.5 and 50 g/L) for 2, 24, and 48 h. *B. aequororis* 5-DB remains metabolically active at pH 7–11 with 0.5 g/L NaCl and pH 7–9 at 50 g/L NaCl (Figure 2). At both salt concentrations, the metabolic activity manifests itself in 2 h of incubation in a medium with pH 5 and 13, but sharply decreases with increasing incubation time. At pH 7–9, activity increases by 24 and 48 h of incubation at 50 and 0.5 g/L NaCl. The metabolic activity of *B. subtilis* ATCC 6633 is also manifested at pH 3 with 0.5 g/L NaCl; however, the specific fluorescence units are significantly lower than those of *B. aequororis* 5-DB (Figure 3). *B. subtilis* ATCC 6633 showed an increase in metabolic intensity with increasing incubation time.

**Figure 4:**
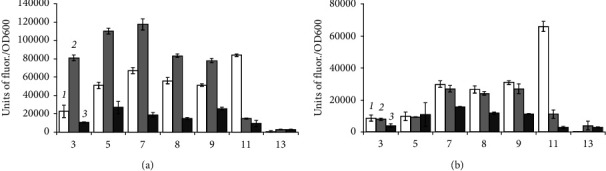
Metabolic intensity (units of fluor./OD600) of *B. aequororis* 5-DB cells from the stationary growth phase after 2 (1), 24 (2), and 48 (3) hours in a medium with 0.5 (a) and 50 (b) g/L NaCl.

**Figure 5:**
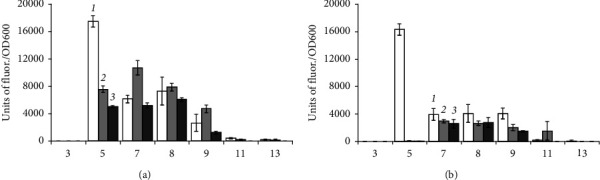
Metabolic intensity (units of fluor./OD600) of *B. subtilis* ATCC 6633 cells from the stationary growth phase after 2 (1), 24 (2), and 48 (3) hours in a medium with 0.5 (a) and 50 (b) g/L NaCl.

*B. aequororis* 5-DB and *B. subtilis* ATCC 6633 cells from the stationary growth phase were incubated in a medium with pH 3–13 with two concentrations of NaCl (0.5 and 50 g/L). The metabolic intensity of *B. aequororis* 5-DB at 0.5 g/L NaCl increases noticeably by the end of the 24-hour incubation period compared to 2-hour, except for pH 11 and 13, but by the end of the 48-hour period the metabolic intensity decreases and becomes lower than the intensity at the 2-hour incubation (Figure 4). Such regularity is not observed at 50 g/L NaCl. The metabolic activity of *B. subtilis* ATCC 6633 cells from the stationary phase of growth at pH 5 increases sharply after 2 h of incubation, but then, by 24 and 48 h, it decreases significantly (Figure 5). The metabolic intensity is low at pH 11 and 13; unlike *B. aequororis* 5-DB, there is no increase of metabolic intensity at the first hours of incubation.

**Figure 6:**
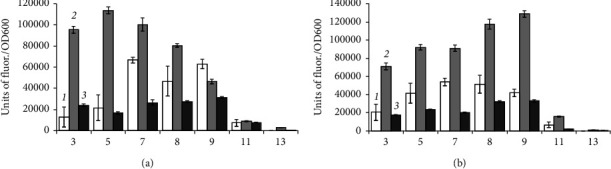
Metabolic intensity (units of fluor./OD600) of *B. aequororis* 5-DB cells from the stationary growth phase after 2 (1), 24 (2), and 48 (3) hours in a medium with 0.5 (a) and 50 (b) g/L NaCl and 1 g/L glucose.

**Figure 7:**
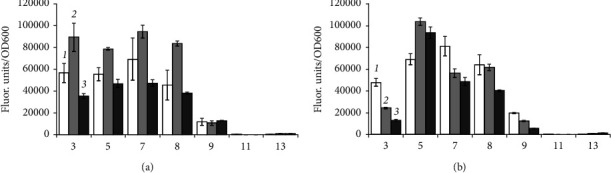
Metabolic intensity (units of fluor./OD600) of *B. subtilis* ATCC 6633 cells from the stationary growth phase after 2 (1), 24 (2), and 48 (3) hours in a medium with 0.5 (a) and 50 (b) g/L NaCl and 1 g/L glucose.

*B. aequororis* 5-DB and *B. subtilis* ATCC 6633 cells were transferred from the stationary growth phase into a medium with pH 3–13 with 0.5 and 50 g/L NaCl and 1 g/L of glucose. The metabolic intensity of *B. aequororis* 5-DB in most cases increases by the end of a 24-hour incubation period of incubation with a further decrease, and this pattern is also observed at 50 g/L NaCl (Figure 6). This trend is observed to a lesser extent in *B. subtilis* ATCC 6633 at 0.5 g/L NaCl, but not at 50 g/L NaCl (Figure 7). The metabolic activity of *B. subtilis* ATCC 6633 is practically absent at pH 11 and 13. Compared to starvation, the metabolic activity of *B. subtilis* ATCC 6633 cells collected from the stationary growth phase in the presence of glucose in the medium significantly increases as shown from the units of fluor./OD600 ratio. An increase in metabolic intensity is also observed at extremely low pH values; however, glucose in the medium does not affect metabolic intensity of *B. subtilis* ATCC 6633 at high pH.

The number of viable cells of *B. aequororis* 5-DB and *B. subtilis* ATCC 6633 from the stationary phase was determined under various pH and mineralization values. CFU/ml of *B. aequororis* 5-DB was shown to decrease by two orders of magnitude at pH 11 and 50 g/L NaCl and to decrease by one order of magnitude after 24 and 48 hours of incubation at pH 5 and 0.5 g/L (S2). *B. subtilis* ATCC 6633 did not form colonies after 48 hours of incubation at pH 5 and pH 11 (0.5 and 50 g/L NaCl), after 24 hours of incubation at pH 5 and pH 11 (50 g/L NaCl) and after 24 and 48 h of incubation at pH 8 and 50 g/L NaCl. After heating the suspension at 80°C for 10 min, *B. aequororis* 5-DB and *B. subtilis* ATCC 6633 did not form colonies.

### 3.2. Assessment of ATP Content

**Figure 8:**
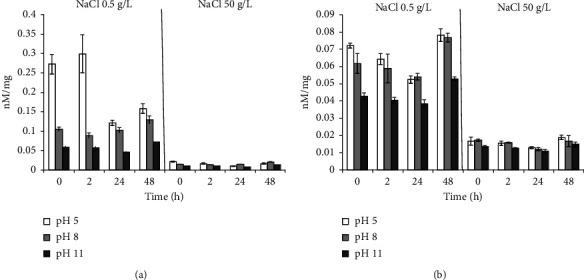
ATP content (nM/mg) in *B. aequororis* 5-DB (a) and *B. subtilis* ATCC 6633 (b) cells from the logarithmic growth phase.

Incubation in a medium with 50 g/L NaCl in all experimental variants for *B. aequororis* 5-DB and *B. subtilis* ATCC 6633 courses to a severalfold decreases in the intracellular ATP content. When *B. aequororis* 5-DB cells are harvested from the logarithmic growth phase and incubated in a medium with pH 5 and 0.5 g/L NaCl, the ATP content exceeds that at pH 8 and 11, but with increasing incubation time, it decreases significantly (Figure 8(a)). The ATP content in *B. subtilis* ATCC 6633 cells is lower than in *B. aequororis* 5-DB cells (Figure 8(b)).

**Figure 9:**
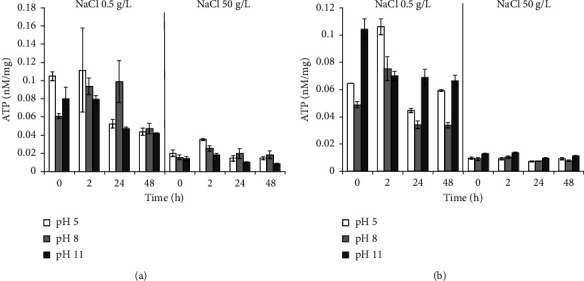
ATP content (nM/mg) in *B. aequororis* 5-DB (a) and *B. subtilis* ATCC 6633 (b) cells from the stationary growth phase.

The ATP content in *B. aequororis* 5-DB and *B. subtilis* ATCC 6633 cells from the stationary growth phase at the initial time in a medium with pH 5 and 11 (0.5 g/L NaCl) was significantly higher than at pH 8, which may be associated with the response to stress. The ATP content in *B. aequororis* 5-DB cells decreased after 24–48 hours of incubation (Figure 9).

**Figure 10:**
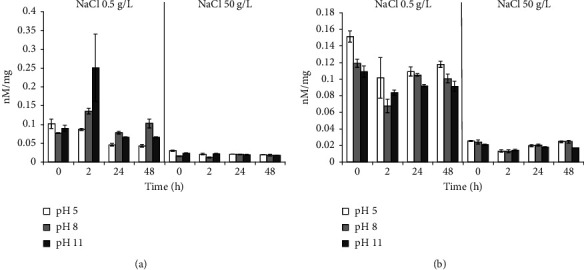
ATP content (nM/mg) in *B. aequororis* 5-DB (a) and *B. subtilis* ATCC 6633 (b) cells from the stationary growth phase with 1 g/L glucose.

Adding 1 g/L glucose to the medium has virtually no effect on *B. aequororis* 5-DB cells (Figure 10(a)) harvested from the stationary growth phase but courses to a significant increase in the ATP content in *B. subtilis* ATCC 6633 cells in unfavorable conditions (pH 5 and 11) (Figure 10(b)). Moreover, if glucose is added to a medium with 50 g/L NaCl, it does not course to an increase in the ATP content in cells.

### 3.3. Assessment of Intracellular pH of *B. aequororis* 5-DB and *B. subtilis* ATCC 6633

The pH_in_ values of the facultative alkaliphile *B. aequororis* 5-DB and the weakly alkali-tolerant *B. subtilis* ATCC 6633 were determined. The pH_in_ was lower than the pH_out_ for both cultures (Table 1). The difference in proton concentrations (ΔpH) in *B. aequororis* 5-DB is higher at pH_out_ 11 (50 g/L NaCl) than at pH_out_ 11 (0.5 g/L NaCl) and at pH_out_ 8 (0.5 and 50 g/L NaCl). *B. aequororis* 5-DB has a wider range of tolerance to pH extremes contrary to *B. subtilis* ATCC 6633. In *B. subtilis* ATCC 6633 cells, ΔpH was zero at pH_out_ 3 (Table 2).

### 3.4. AFM and Phase-Contrast Microscopy of *B. aequororis* 5-DB and *B. subtilis* ATCC 6633 Cells

**Figure 11:**
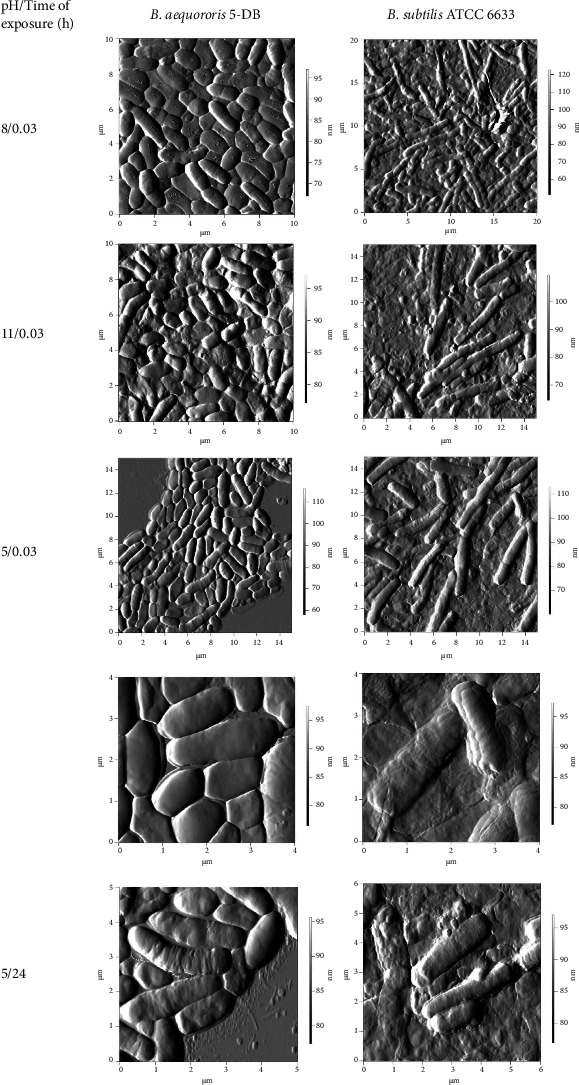
AFM images of *B. aequororis* 5-DB and *B. subtilis* ATCC 6633 cells.

We studied the effect of low and high pH on the morphology of alkaliphilic *B. aequororis* 5-DB and weakly alkali-tolerant *B. subtilis* ATCC 6633 cells (Figure 11). Morphology changes of cells were investigated immediately and after 24 h of adaptation. Cell lysis and cell debris were observed after 24 h of incubation of *B. subtilis* ATCC 6633 cells in the medium with pH 5. On the contrary, *B. aequororis* 5-DB was more resistant to pH 5; the cells were even and had a smooth surface after a short exposure in that kind of medium. The surface roughness increased after 24 h of exposure, but a cell lysis was insignificant.

The morphometric parameters (length, width, height, and surface roughness) of bacilli cells were assessed after incubation in the medium with pH 5 and 11. The control conditions were the same as those of cultivation (0.5 g/L NaCl and pH 11 for *B. aequororis* 5-DB; 0.5 g/L NaCl and pH 8 for *B. subtilis* ATCC 6633). The surface roughness of *B. aequororis* 5-DB did not differ significantly from the control when cells were incubated in an acidic medium (pH 5). In this case, the cell volume increased by 40% (Table 3). During incubation in this medium for 24 hours, a slight increase in roughness and a significant elongation of cells were observed. The roughness of *B. subtilis* ATCC 6633 cells increased by 31–35% under pH 5, regardless of the time of incubation in this media. Slight shortening of cells and an increase in transverse dimensions relative to the control were also observed.

Images of cells of both strains incubated at pH 5, 8, 11, and 0.5 and 50 g/L salt for 2, 24, and 48 h were obtained by phase-contrast microscopy and presented in S1. When incubated with 50 g/L salt, *B. aequororis* 5-DB showed the appearance of single opalescent cells, presumably spores. Sporulation of *B. subtilis* ATCC 6633 was not observed under the studied conditions.

## 4. Discussion

*B. aequororis* 5-DB was isolated from an alkaline highly mineralized medium from the soil surface of a soda sludge storage in the rich medium with pH 11 [11]. The strain grew very poorly in the rich medium with pH 8 and was classified as a facultative alkaliphile. Its adaptive reactions were considered in comparison with the weakly alkali-tolerant *B. subtilis* ATCC 6633. The following methods were chosen for comprehensive assessment of adaptive reactions under various pH values and 50 g/L NaCl. The metabolic state of the cell was assessed by PrestoBlue HS Cell Viability Reagent dye. The dye contains resazurin, which is reduced to fluorescent resorufin by dehydrogenases in living cells. ATP content was assessed using the bioluminescent method. Viability, number of spores, and vegetative cells were assessed by CFU/mL. Intracellular pH was assessed by fluorescent cFDASE probe. Bacterial cells were visualized by AFM and phase-contrast light microscopy.

Metabolic activity at different pH and high mineralization was assessed in three options. First and second options: cells were obtained from (1) logarithmic growth phase and (2) stationary growth phase and placed in a buffer with different pH values and 0.5 or 50 g/L NaCl. Third option: cells were obtained from the stationary growth phase and placed in a buffer with different pH values, 0.5 or 50 g/L NaCl, and 1 g/L glucose. Alkaliphiles are known to exhibit multiple adaptive responses that allow them to live at high pH [7]. When growing in an alkaline medium characterized by a low content of protons and a high content of sodium ions, in addition to osmoadaptation, microorganisms need to maintain intracellular pH homeostasis. Protons removed from the cell during respiration are not able to do useful work when they return to the cytoplasm, since they will move against the concentration gradient. Replacement of sodium ions by protons is carried out by Na^+^/H^+^ antiporters. Formation of chemiosmotic reverse ΔpH is provided by a Na^+^ electrochemical gradient. A major strategy for bacterial pH homeostasis is the use of transporters that catalyze active proton transport. These transporters include primary proton pumps, such as proton pumping respiratory chain complexes or proton coupled ATPases, and secondary active transporters, such as cation-proton antiporters, which use the proton motive force (PMF), generated by respiration or ATPases to energize active uptake of protons in exchange for cytoplasmic cations, such as Na^+^ or K^+^ [8, 26]. In the stationary phase of growth, bacteria are already preadapted to adverse changes, since in this phase, RpoS becomes the main subunit of RNA polymerase [27].

We compared the adaptive response of the alkaliphilic *B. aequororis* 5-DB and the weakly alkali-tolerant *B. subtilis* ATCC 6633 in different growth phases. It was shown that the metabolic intensity of *B. aequororis* 5-DB cells harvested from the log phase of growth increases with increasing exposure time under a pH value different from the pH of the culture medium, while the metabolic intensity of cells harvested from the stationary phase of growth decreases after 48 hours of exposition. The presence of glucose in the medium allowed *B. aequororis* 5-DB to significantly increase its metabolic intensity after 24 hours of incubation at 50 g/L NaCl, although the strain did not utilize glucose as a carbon source. In almost all the variants of the experiment, metabolic intensity increases after 24 hours of cell adaptation under these pH conditions. Despite the fact that *B. aequororis* 5-DB was grown at pH 11, only cells harvested from the log growth phase exhibit high metabolic intensity under these conditions. In the active growth phase, the cell at pH 11 is able to receive energy and function actively due to the Na^+^/H^+^ antiporter, while in the stationary phase, after the growth substrate is depleted, its metabolic activity decreases. *B. subtilis* ATCC 6633 cells harvested from the stationary growth phase showed metabolic activity at pH 3 and 5 in the presence of glucose. *B. subtilis* ATCC 6633, unlike *B. aequororis* 5-DB, utilizes glucose, which allows this bacterium to adapt to low pH. The effect of glucose on the adaptation of *B. subtilis* ATCC 6633 is confirmed by data on ATP content. The addition of 1 g/L glucose to the medium has virtually no effect on the ATP content in *B. aequororis* 5-DB cells taken from the stationary growth phase, but courses to a significant increase in the ATP content in *B. subtilis* ATCC 6633 cells even at pH 5 and 11. These data are consistent with the fact that *B. subtilis* ATCC 6633 grows on glucose, while *B. aequororis* 5-DB does not utilize glucose. *B. aequororis* 5-DB cells harvested from the stationary growth phase become more adapted to low pH, and the strain exhibits metabolic activity at pH 3 and 5, while the activity was absent in cells from the logarithmic growth phase. The ATP content in *B. subtilis* ATCC 6633 cells from the stationary growth phase under low and high pH at 0.5 g/L approaches that at optimal pH and, in some cases, even exceeds it. The ATP content in the cells of both *B. aequororis* 5-DB and *B. subtilis* ATCC 6633 from the logarithmic growth phase at pH 5 and 0.5 g/L NaCl is significantly higher than at pH 11. Accumulation of ATP in the cell may result from the uncoupling of constructive and energy metabolism at low pH, which leads to the accumulation of ATP in the cell. In this case, cell death does not occur.

Statistical analysis showed significant differences (Student's *t*-test, *p* < 0.05) of the metabolic intensity of *B. aequororis* 5-DB cells from the logarithmic growth phase under all pH and 0.5 g/L salt in the medium from that under pH 7. At 50 g/L salt in the medium, there is a significant difference only under extreme pH (3, 5, 11, and 13). For *B. subtilis* ATCC 6633, the differences are significant in the first 2 hours of incubation; the metabolic intensity is also significantly different at extreme pH from pH 7. In *B. aequororis* 5-DB cells from the stationary phase of growth, a significant increase in metabolic intensity is observed at 24 hours compared to 2 hours of incubation. At 48 hours, there is a significant decrease in metabolic intensity both with the addition of glucose and under starvation conditions. In B. subtilis ATCC 6633 cells from the stationary growth phase, a significant decrease in the metabolic rate was observed at pH >8 compared to pH 7. A significant decrease in the intracellular ATP content was shown in both *B. aequororis* 5-DB and *B. subtilis* ATCC 6633 at 50 g/L NaCl compared to 0.5 g/L.

The difference between pH_in_ and pH_out_ in *B. aequororis* 5-DB is maximal at pH 11 with 0.5 and 50 g/L NaCl and is not equal to 0 at pH 3. It was noted that cells of *B. subtilis* ATCC 6633 at pH 3 are not viable, and pH_in_ equals to pH_out_. The pH_in_ value of *B. subtilis* ATCC 6633 is higher than the pH_in_ value of *B. aequororis* 5-DB at pH_out_ 11. It is known that the Na^+^/H^+^ antiporter plays the major role in the pH homeostasis of alkaliphiles [28]; therefore, bacteria need Na^+^ ions for their vital activity. We have shown that the maximum ΔpH is observed at pH 11 and 50 g/L NaCl in both *B. aequororis* 5-DB and *B. subtilis* ATCC 6633. High concentration of Na^+^ in the medium coupled with high pH_out_ course to activating Na^+^/H^+^ antiporter. Na^+^ enters the cell and is exchanged for H+ during antiporter's work, which leads to an increase in the pH gradient. In addition, other Na^+^ transporters are known. Comparison of the whole-genome sequence of *B. halodurans* C-125 with that of *B. subtilis* revealed that in addition to the F_1_F_0_-ATP synthase operon, an operon for a Na^+^-transporting ATP synthase is also revealed in both *B. halodurans* C-125 and *B. subtilis* [9]. Strong alkalization of the cytoplasm (up to pH 9) is observed in *B. aequororis* 5-DB after 48 h. This correlates with a decrease in the metabolic activity of cells after 48 h of incubation in this medium.

Sporulation plays an important role in the adaptation of bacilli [29]. *B. subtilis* is able to grow at a pH of 4.8 to 9.2 and a water activity above 0.929. Gauvry et al. reported that the transition from favorable conditions to unfavorable conditions results in a delay and even a cessation of spore formation, while in favorable conditions, the sporulation process resumes. This can be explained by the fact that low pH courses to inhibition of the expression of sporulation genes and slows down enzymatic reactions. Vegetative cells sporulate more synchronously, and in higher proportions under suboptimal conditions for sporulation, the level of phosphorylated Spo0A increases to a high level in stress cells, which allows of more efficient initiation of the sporulation process [30]. It was shown that lower temperatures and pH slowed down the sporulation process [31]. Although *B. aequororis* [12] and *B. subtilis* [32] are the sporulating bacteria, we showed that exposure to the conditions studied did not result in active sporulation in the bacilli. In our opinion, extreme pH values and high salt concentrations did not cause active sporulation, firstly, because these conditions were not suboptimal but critical for the cells. Secondly, the cells were incubated in a medium with different pH values and mineralization in the absence of a nutrient substrate, which negatively affected all metabolic processes in the cell and gene expression, including sporulation genes.

According to the results of AFM, the cells of the alkaliphile *B. aequororis* 5-DB are less damaged in the medium with low pH than the weakly alkali-tolerant *B. subtilis* ATCC 6633. Under low pH, the roughness of *B. subtilis* ATCC 6633 cell surface increases relative to the control (pH 8) by 36%. Under low pH, the surface roughness of *B. aequororis* 5-DB cells increases relative to the control (pH 11) by 26%. An increase in surface roughness indicates a decrease in cell turgor. Low pH has little effect on cell size in both strains.

The limitations of this study are the experimental conditions, namely, exposure to high pH and mineralization under starvation conditions, while conditions close to the natural habitat will expand our understanding of the adaptation of alkaliphilic bacilli. Furthermore, research directions may concern changes in the conditions of exposure to pH and mineralization and analysis of gene expression under such effects.

Thus, an interesting fact is the increased resistance of the facultative alkaliphile *B. aequororis* 5-DB to low pH, which confirms the general nonspecific adaptive ability of this strain to adverse environmental conditions. We previously reported that *B. aequororis* 5-DB exhibits lipase and amylase activities under conditions of high alkalinity and mineralization [13]. This strain can be used in feed production and in the production of agricultural probiotics and enzymes included in detergents, and processing of biogenic raw materials and waste from the food industry and agriculture.

## 5. Conclusion

Based on the proposed methods, we assessed the effect of changes in pH and mineralization of the medium on the metabolic intensity, morphology, and pH_in_ of cells of two strains of alkaliphilic and weakly alkali-tolerant bacilli. We showed a significantly greater resistance of alkaliphilic *B. aequororis* 5-DB not only to high but also to low pH in comparison with *B. subtilis* ATCC 6633. The resistance of *B. aequororis* 5-DB to low pH values was expressed in higher metabolic intensity, estimated by reduction of resazurin to resorufin by dehydrogenases, maintenance of ΔpH, no significant cell damage, and slight increase in surface roughness. Alkaliphilic *B. aequororis* 5-DB has a broader general resistance to adverse factors (wide pH range, 50 g/L NaCl) than a weakly alkali-tolerant strain of the same genus. Such nonspecific resistance may be promising for the biotechnological application of *B. aequororis* 5-DB.

## Figures and Tables

**Table 1:** PH_in_ and ΔpH in *B. aequororis* 5-DB with different pH_out_ and salt concentrations.

NaCl (g/L)	рН_out_	pH_in_	|ΔpH|	pH_in_	|ΔpH|
		2 h		48 h	
0.5	3	4.4	1.4	4.4	1.4
	8	7.7	0.3	7.4	0.6
	9	7	2	8.2	0.8
	11	8.6	2.4	9.3	1.7

50	3	3.9	0.9	3	0
	8	7	1	7.2	0.8
	9	6.8	2.2	8	1
	11	7.9	3.1	9.1	1.9

**Table 2:** PH_in_ and ΔpH in *B. subtilis* ATCC 6633 with different pH_out_ and salt concentrations.

NaCl, g/L	рН_out_	pH_in_	|ΔpH|	pH_in_	|ΔpH|
		2 h		48 h	
0.5	3	3	0	3	0
	8	7.6	0.4	7.5	0.5
	9	7.6	1.4	7.8	1.2
	11	9.1	1.9	8.3	2.7

50	3	3	0	3	0
	8	7.7	0.3	7.5	0.5
	9	7.6	1.4	7.9	1.1
	11	8.2	2.8	8.1	2.9

**Table 3:** Morphometric parameters of *B. aequororis* 5-DB and *B. subtilis* ATCC 6633 cells.

рН/time of exposure (h)	Roughness (nm)	Length (*µ*m)	Width (*µ*m)	Height (*µ*m)	*V* (*µ*m^3^)
*B. aequororis 5-DB*					
8/0.03	104.95	1.33 ± 0.08	0.97 ± 0.01	0.23 ± 0.01	0.16
11/0.03	75.00	1.79 ± 0.02	1.15 ± 0.31	0.23 ± 0.02	0.25
5/0.03	81.53	2.10 ± 0.19	1.07 ± 0.07	0.30 ± 0,01	0.35
5/24	95.36	2.87 ± 0.05	0.78 ± 0.04	0.21 ± 0.01	0.25

*B. subtilis ATCC 6633*					
8/0.03	77.35	3.97 ± 0.05	0.74 ± 0.02	0.27 ± 0.01	0.42
11/0.03	96.98	4.28 ± 0.39	1.54 ± 0.22	0.20 ± 0.01	0.69
5/0.03	105.80	3.38 ± 0.04	0.94 ± 0.02	0.29 ± 0.04	0.48
5/24	101.91	3.23 ± 0.13	0.82 ± 0.09	0.31 ± 0.01	0.43

## Data Availability

The datasets generated and/or analyzed during the current study are available from the corresponding author upon reasonable request.

## Supplementary Materials

Supplementary Materials Supplementary materials S1: morphology of *B*. *aequororis* 5-DB and *B. subtilis* ATCC 6633 cells under various pH and high mineralization of the medium (phase-contrast microscopy). Supplementary materials S2: the content of viable cells of *B. aequororis* 5-DB and *B. subtilis* ATCC 6633 under various pH and mineralization of the medium (CFU/ml).
