# Supplementary material for: Metabolic and Morphological Aspects of Adaptation of Alkaliphilic Bacillus aequororis 5-DB and Alkali-Tolerant Bacillus subtilis ATCC 6633 to Changes in pH and Mineralization
Source: Int J Microbiol. 2024 Jul 23;2024:3087296. doi: 10.1155/2024/3087296 (PMC11288695; doi:10.1155/2024/3087296)
Supplement: Supplementary Materials — S1: morphology of B. aequororis 5-DB and B. subtilis ATCC 6633 cells under various pH and high mineralization of the medium (phase-contrast microscopy). Supplementary materials S2: the content of viable cells of B. aequororis 5-DB and B. subtilis ATCC 6633 under various pH and mineralization of the medium (CFU/ml). [file 3087296.f1.pdf]

SUPPLEMENTARY MATERIALS

S1. Morphology of *Bacillus aequororis* 5-DB and *Bacillus subtilis* ATCC 6633 cells under various pH and high mineralization of the medium (phase contrast microscopy).

Morphology of *B. aequororis* 5-DB cells under various pH, mineralization of the medium, and incubation time (h) was assessed by phase contrast microscopy. Arrows indicate spores that are opalescent in phase contrast.

<table>
<tr><td colspan="4">B. aequororis 5-DB</td></tr>
<tr><td colspan="2">Parent culture</td><td colspan="2">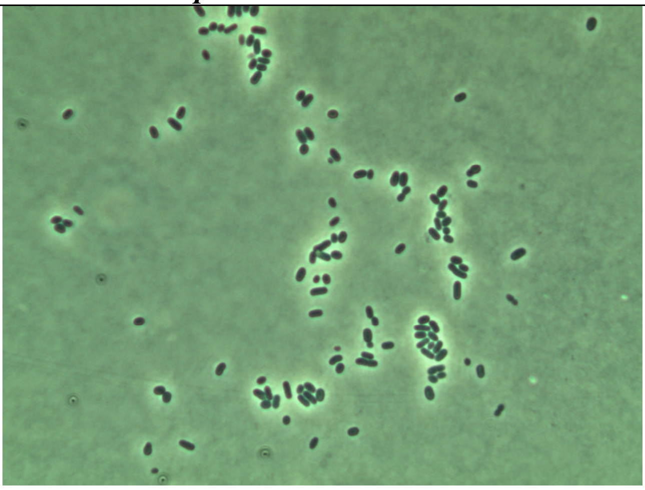</td></tr>
<tr><td rowspan="2">pH</td><td rowspan="2">h</td><td colspan="2">NaCl, g/L</td></tr>
<tr><td>0.5</td><td>50</td></tr>
<tr><td>5</td><td>2</td><td>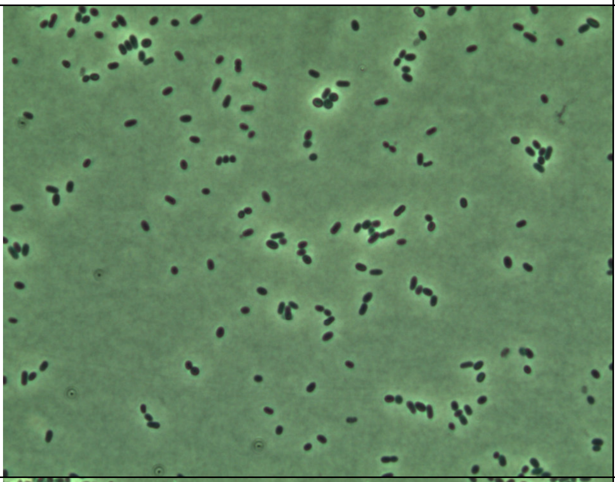</td><td>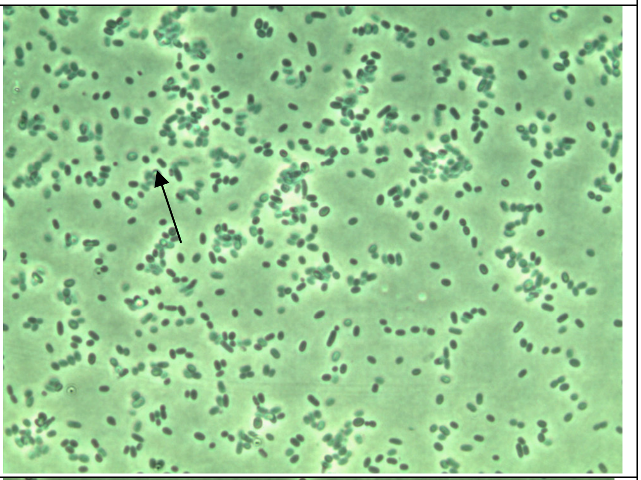</td></tr>
<tr><td>5</td><td>24</td><td>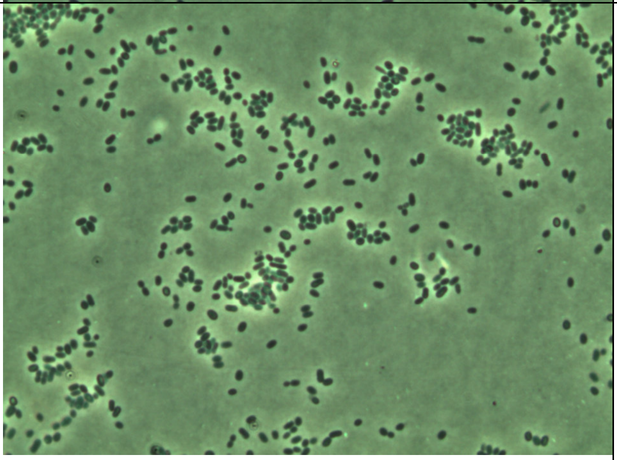</td><td>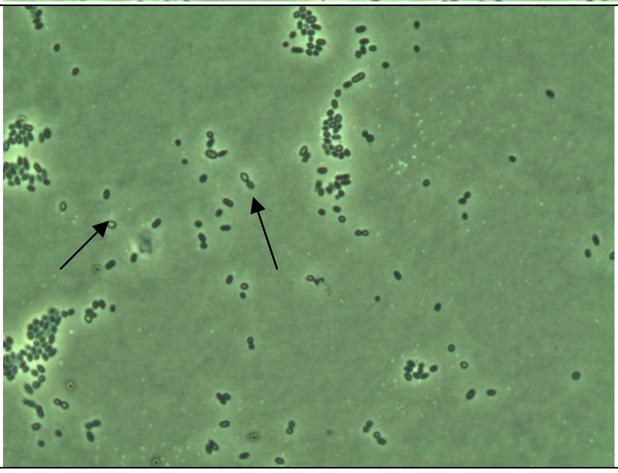</td></tr>
</table>

**5**	**48**	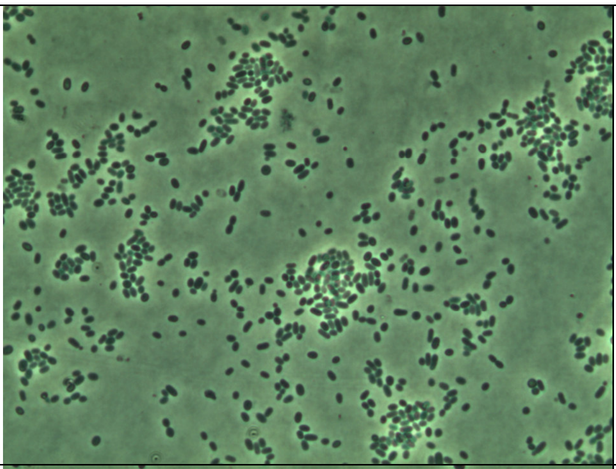	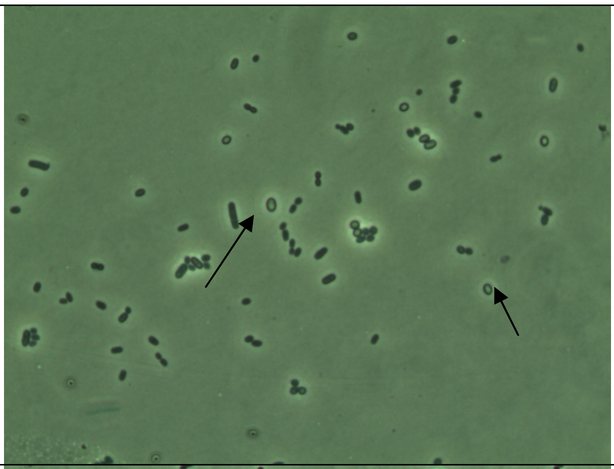
**8**	**2**	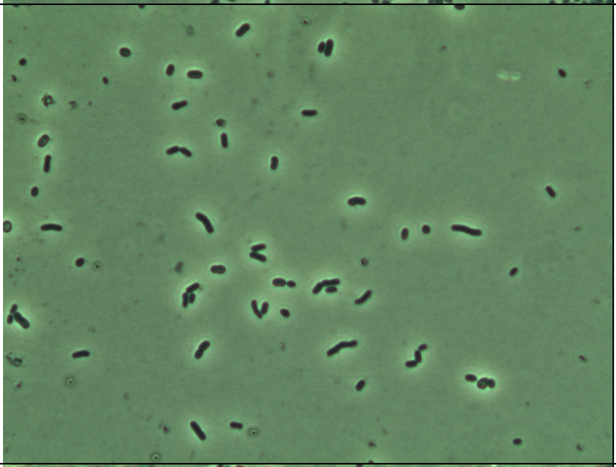	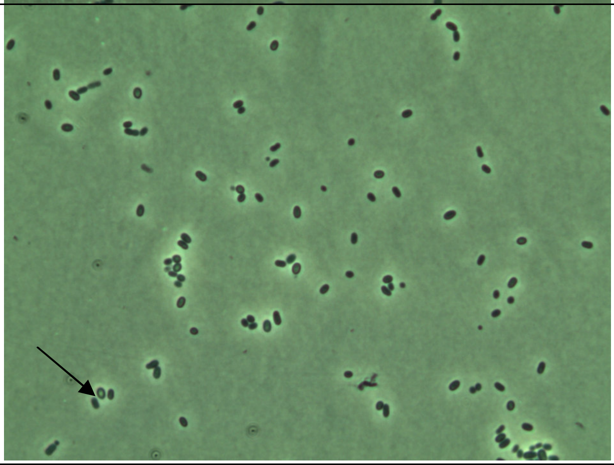
**8**	**24**	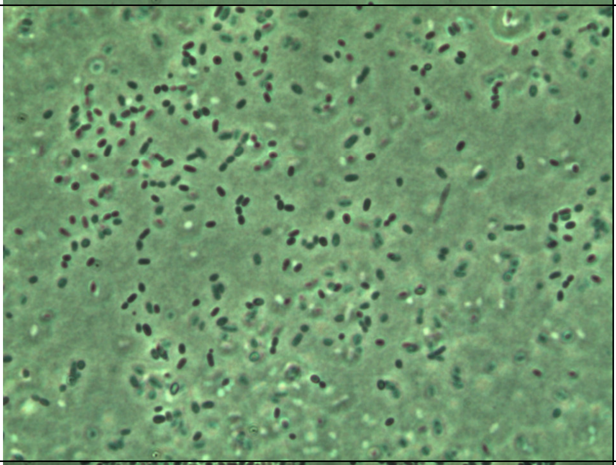	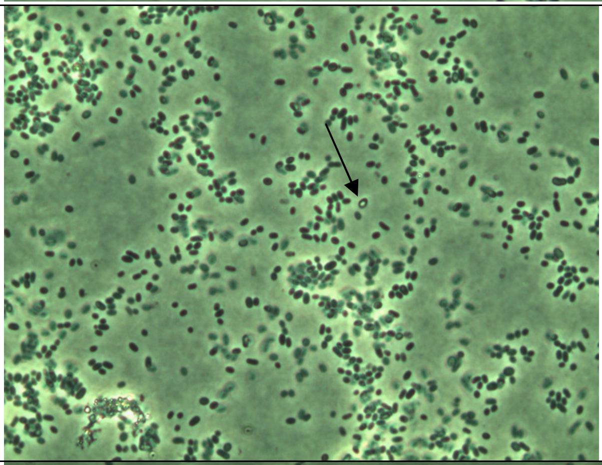
**8**	**48**	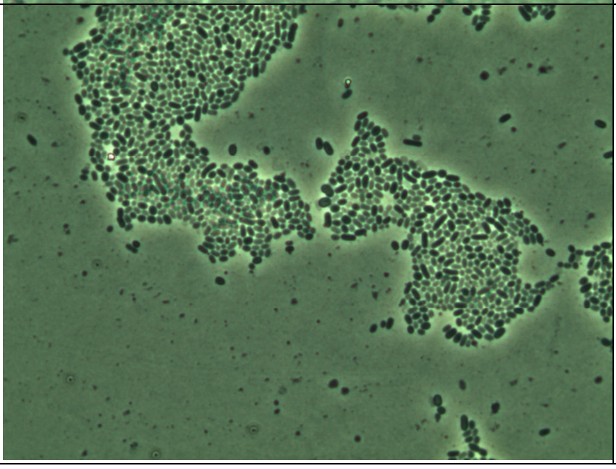	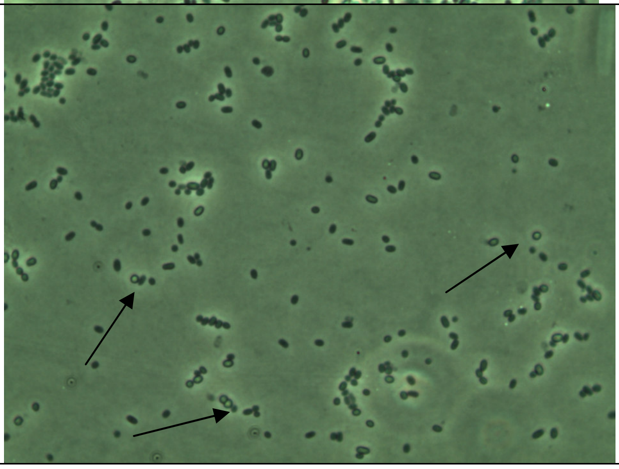

**11**	**2**	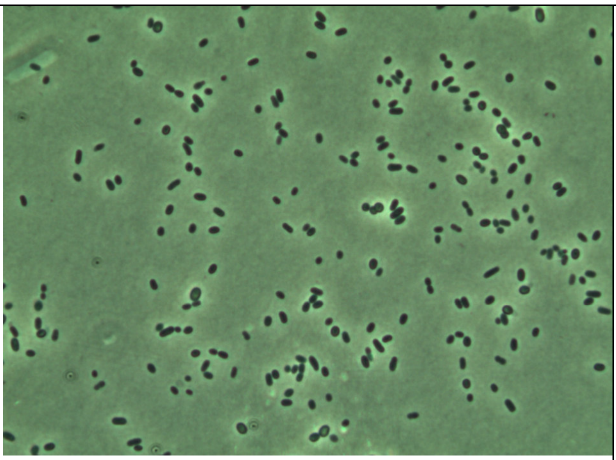	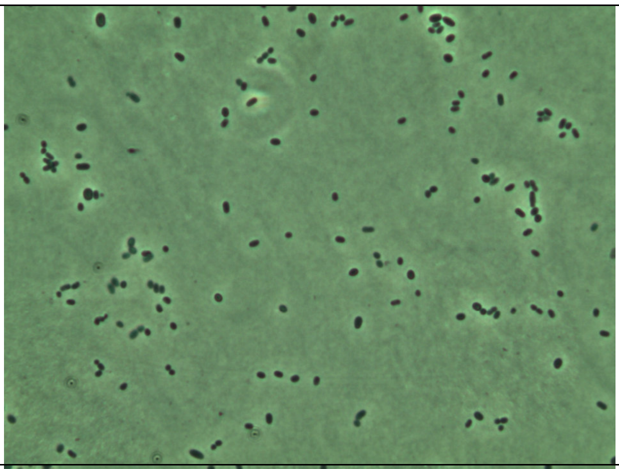
**11**	**24**	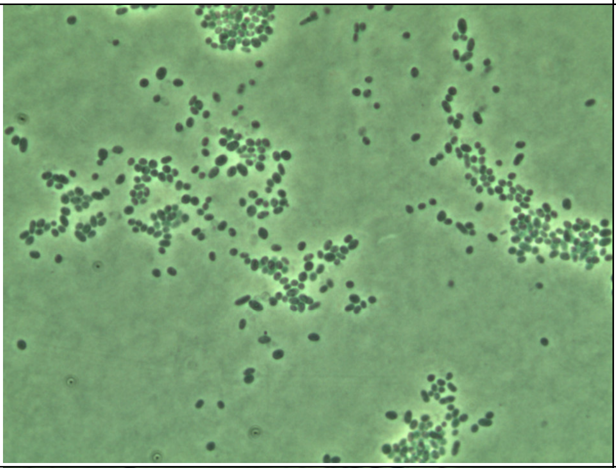	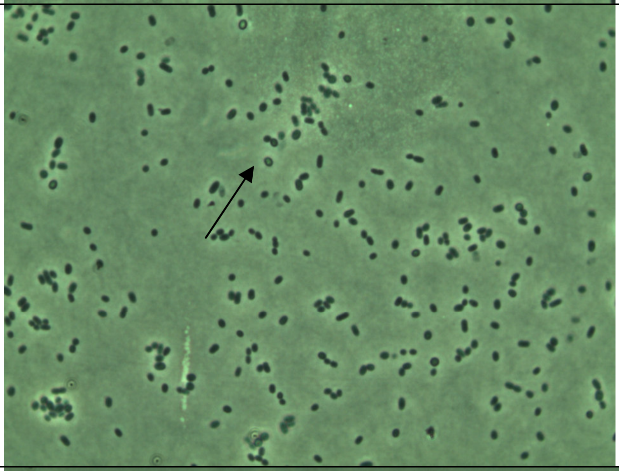
**11**	**48**	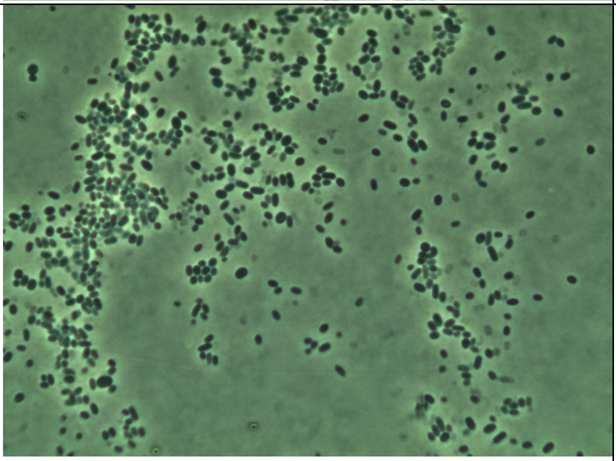	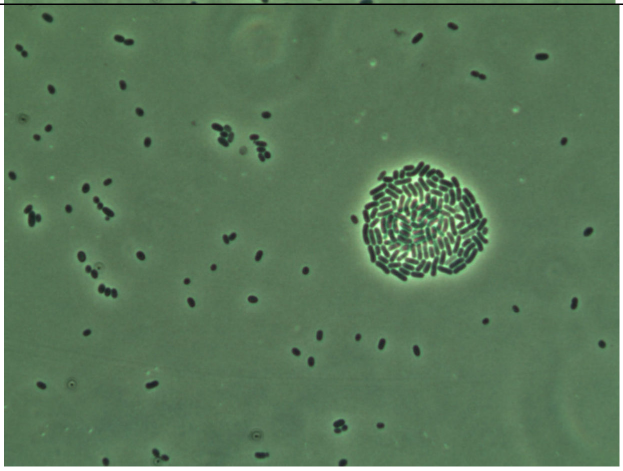
***B. subtilis* ATCC 6633**			
Parent culture		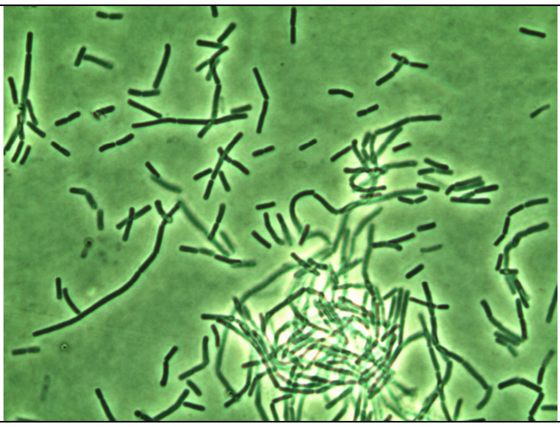	

pH	h	NaCl, g/L	
		**0.5**	**50**
**5**	**2**	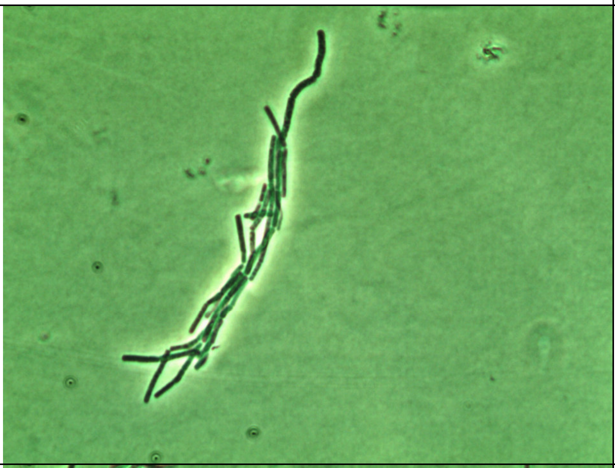	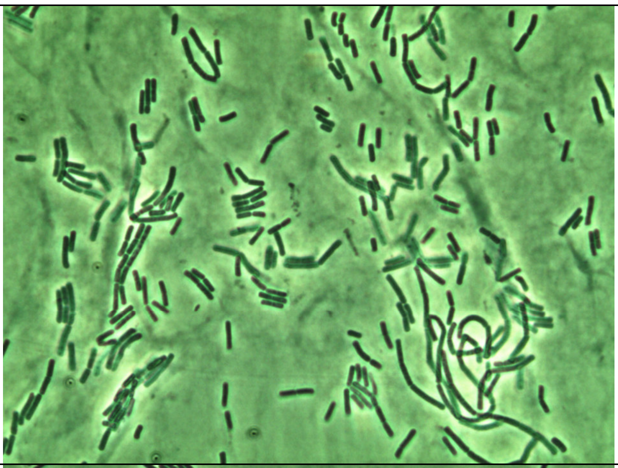
**5**	**24**	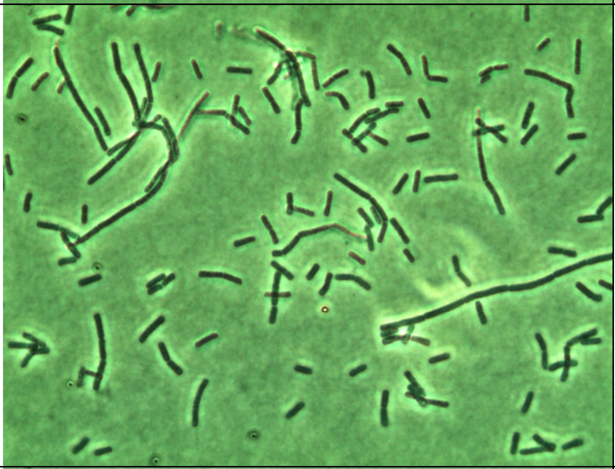	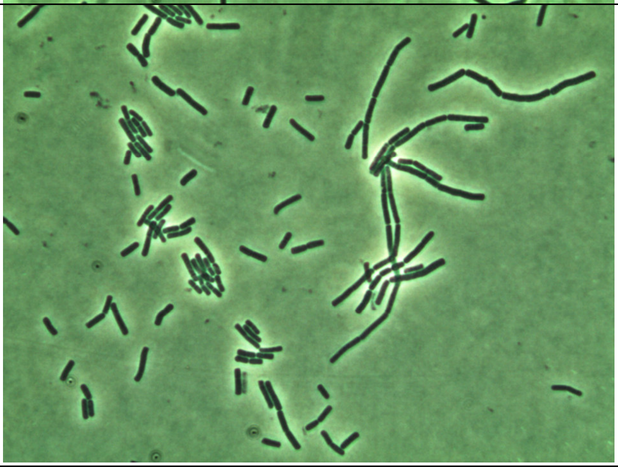
**5**	**48**	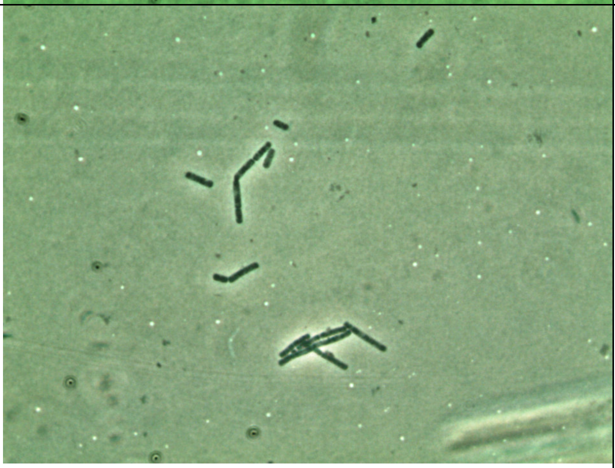	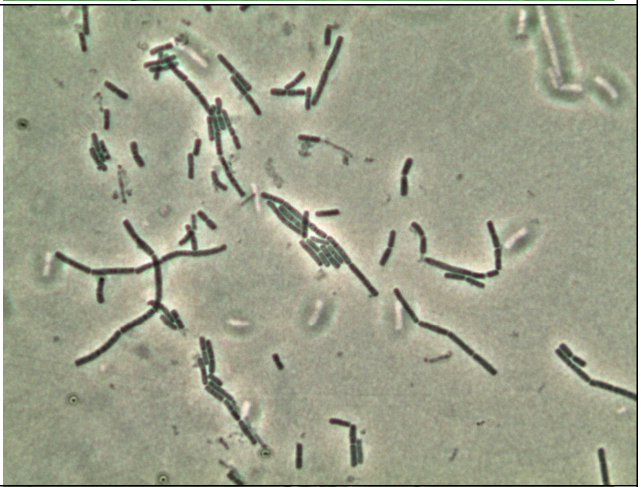
**8**	**2**	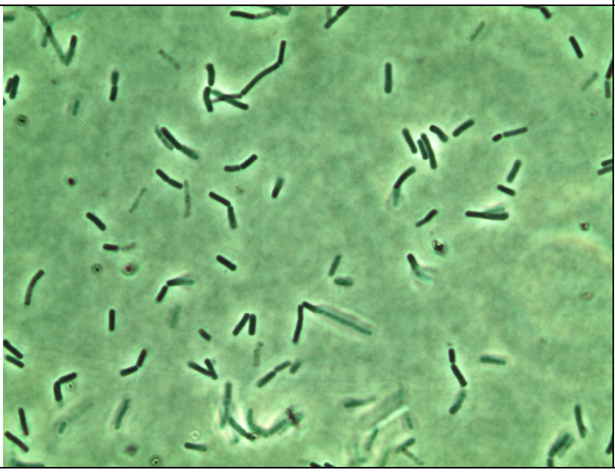	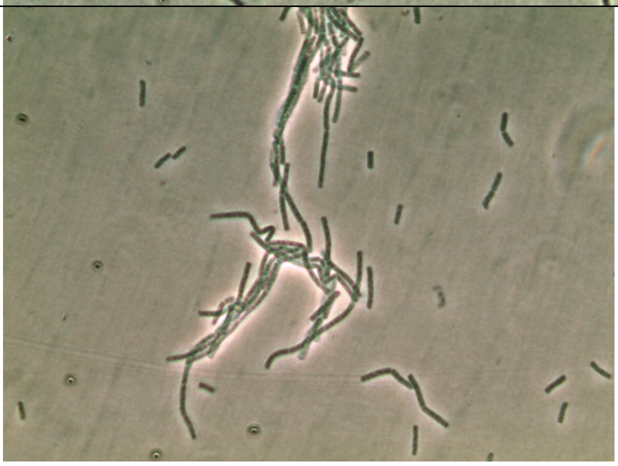

**8**	**24**	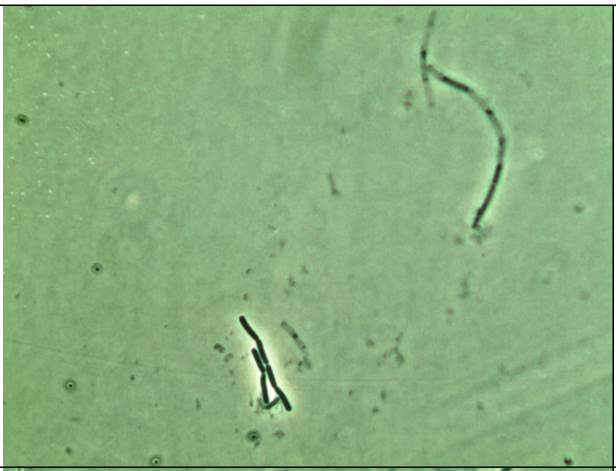	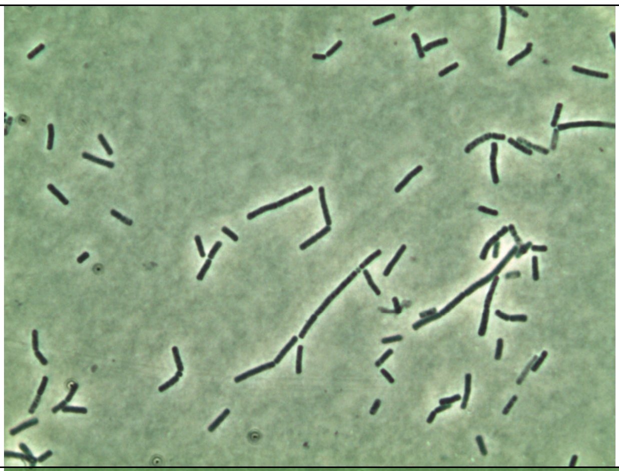
**8**	**48**	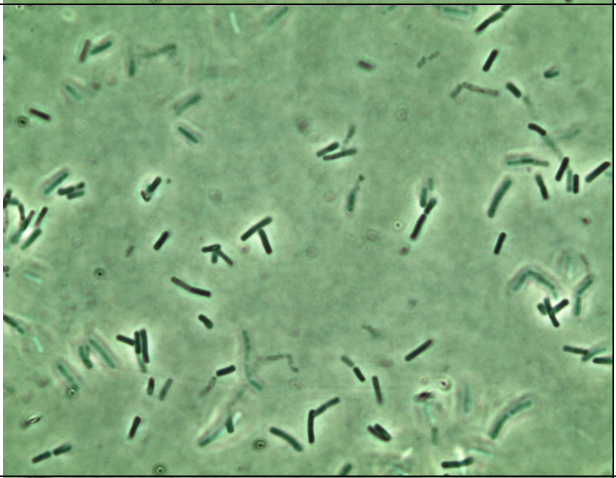	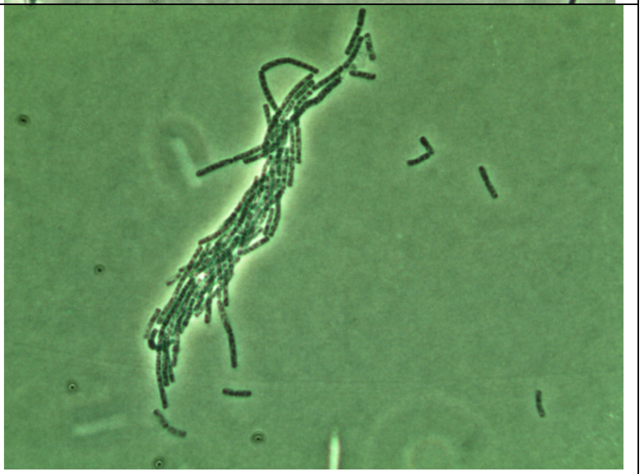
**11**	**2**	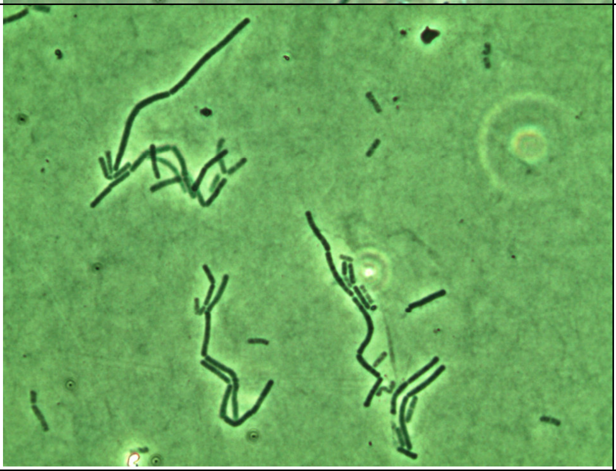	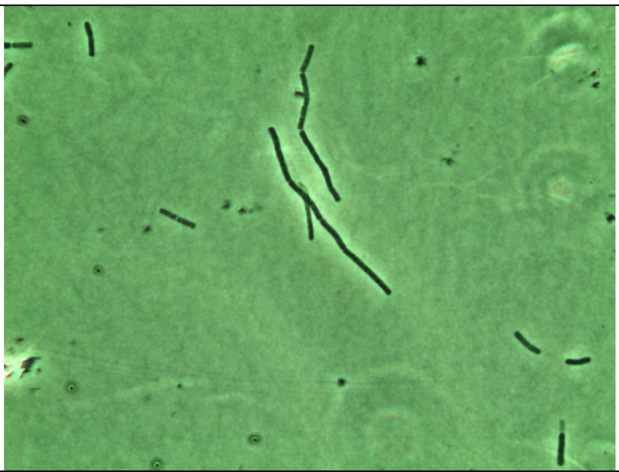
**11**	**24**	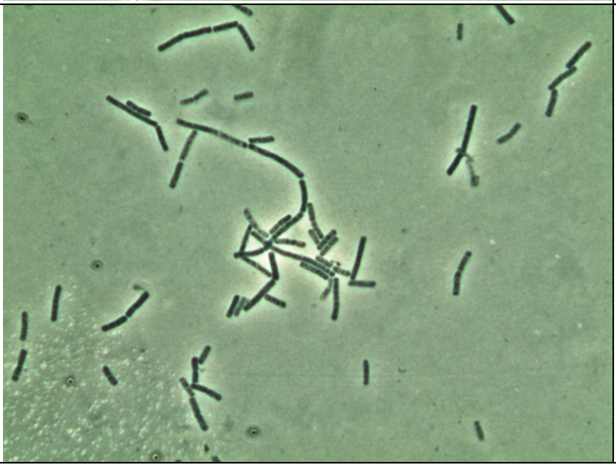	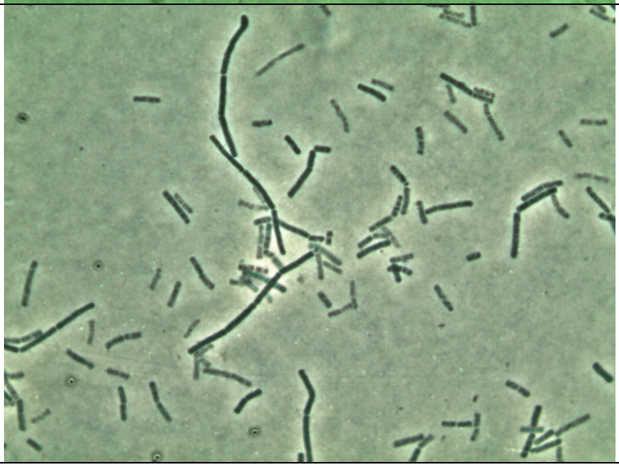

**11**	**48**	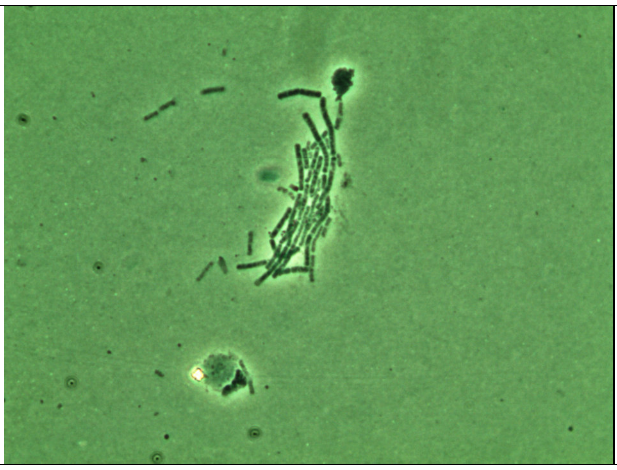	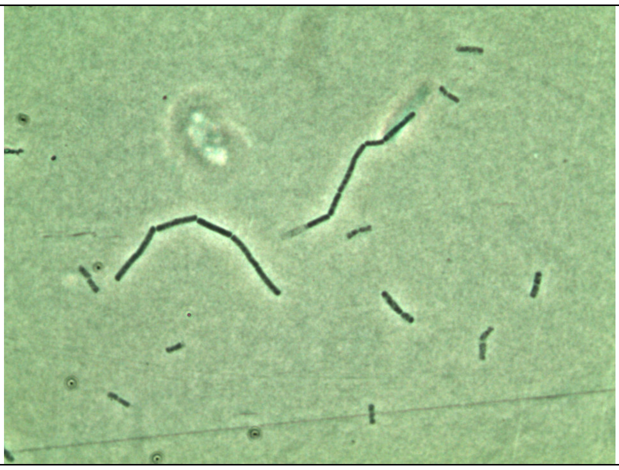

S2. The content of viable cells of *B. aequororis* 5-DB and *B. subtilis* ATCC 6633 under various pH and mineralization of the medium (CFU/ml)

The content of viable cells of *B. aequororis* 5-DB and *B. subtilis* ATCC 6633 was assessed under different pH and mineralization of the medium.

Time of exposure, h	*B. aequororis* 5-DB		*B. subtilis* ATCC 6633	
0	$(3.00 \pm 0.55) \times 10^7$		$(1.85 \pm 0.30) \times 10^6$	
	0.5	50	0.5	50
**pH 5**				
2	$(1.40 \pm 0.21) \times 10^7$	$(1.73 \pm 0.27) \times 10^7$	$(1.25 \pm 0.77) \times 10^5$	$(1.00E \pm 0.80) \times 10^5$
24	$(7.00 \pm 0.74) \times 10^6$	$(1.60 \pm 0.20) \times 10^7$	$(5.00 \pm 1.33) \times 10^5$	0
48	$(5.50 \pm 0.93) \times 10^6$	$(1.55 \pm 0.24) \times 10^7$	0	0
**pH 8**				
2	$(2.33 \pm 0.35) \times 10^7$	$(1.94 \pm 0.38) \times 10^7$	$(1.18 \pm 0.18) \times 10^6$	$(1.25 \pm 0.77) \times 10^5$
24	$(2.25 \pm 0.33) \times 10^7$	$(1.85 \pm 0.27) \times 10^7$	$(7.00 \pm 0.60) \times 10^5$	0
48	$(1.98 \pm 0.28) \times 10^7$	$(1.88 \pm 0.27) \times 10^7$	$(4.75 \pm 0.86) \times 10^5$	0
**pH 11**				
2	$(1.60 \pm 0.21) \times 10^7$	$(4.50 \pm 0.86) \times 10^5$	$(2.75 \pm 0.58) \times 10^5$	$(7.50 \pm 6.78) \times 10^3$
24	$(1.60 \pm 0.23) \times 10^7$	$(2.10 \pm 0.38) \times 10^5$	$(1.50 \pm 0.73) \times 10^5$	0
48	$(1.60 \pm 0.36) \times 10^7$	$(1.90 \pm 0.34) \times 10^5$	0	0
